# Look, over there! A streaker! – Qualitative study examining streaking as a behaviour change technique for habit formation in recreational runners

**DOI:** 10.1080/21642850.2024.2416505

**Published:** 2024-10-21

**Authors:** Meghan Curran, Nicholas Larade, Gözde Özakinci, Gabriela Tymowski-Gionet, Stephan U. Dombrowski

**Affiliations:** a Faculty of Kinesiology, University of New Brunswick, Fredericton, Canada; b Division of Psychology, University of Stirling, Stirling, Scotland

**Keywords:** Streaking, physical activity, running, habit theory, behaviour change techniques

## Abstract

**Background:**

Running as a form of physical activity is beneficial to overall health and wellbeing. The aim of the study is to examine ‘run streaking’ (i.e. running on consecutive days, for a minimum period of time or distance, typically at least one mile) as a technique for habit formation and behaviour change.

**Methods:**

Qualitative semi-structured interviews with 21 recreational adult runners (11 female and 10 male). Run streak length ranged from a minimum of 100 days to over 4500 days. Transcripts were analysed using a hybrid deductive–inductive thematic analysis.

**Results:**

Run streaking was reported to lead to several benefits, health improvements and a sense of accomplishment, although many run streakers reported running through injuries and lack of recovery. Accounts of run streaking showed features of automaticity indicative of habitual behaviour. Other behavioural processes identified included motivation, identity, self-regulation and social support. Behavioural streaking showed the potential to influence change in behaviours other than running.

**Conclusion:**

Accounts of run streaking demonstrate an interplay between automatic and deliberate processes in the maintenance of running behaviour. Behavioural streaking is a technique that could be used in other behaviour change contexts beyond running to support habit formation.

## Introduction

Regular physical activity is beneficial for physical and mental health and reduces mortality (Posadzki et al., 2020; Warburton & Bredin, 2017). Current World Health Organisation guidelines recommend that all adults aged 18–64 engage in 150–300 min of moderate-intensity or 75–150 minutes of vigorous-intensity physical activity per week, or an equivalent combination (Bull et al., 2020). The Canadian 24-hour movement guidelines recommend that adults aged 18–64 engage in at least 150 minutes of moderate-to-vigorous-intensity physical activity per week and be physically active on a daily basis (Ross et al., 2020). Higher levels of physical activity typically increase health benefits, with added benefits diminishing somewhat at very high volumes (Bull et al., 2020; Janssen & Ross, 2012; Lee et al., 2022; Wen et al., 2011).

Many individuals are inactive, despite evidence on the benefits of physical activity. A pooled analysis of 358 population-based surveys with 1.9 million participants estimated that globally over one quarter of adults (27.5% of the world’s adult population) (Guthold et al., 2018) are insufficiently physically active. Rates of inactivity were stable over time, higher in women and high-income Western countries. In Canada, for example, using accelerometer-measured physical activity, it has been estimated that adults engage in 26 minutes of moderate-to-vigorous-intensity physical activity per day, of which four minutes are at the vigorous intensity level (Clarke et al., 2019). Increasing levels of physical activity, particularly at vigorous-intensity level, would lead to substantial health benefits.

Recreational running is one form of vigorous-intensity physical activity, with evidence of health benefits (Chakravarty et al., 2008; Kozlovskaia et al., 2019; Lee et al., 2014; Oja et al., 2017; Pedisic et al., 2020). Running is one of the most popular global sports and leisure time physical activities (Hulteen et al., 2017) and has several advantages compared to other activities, including that it is inexpensive, can be performed in a variety of contexts, and across different ages, genders, health statuses and ethnicities, thereby overcoming many common barriers to physical activity engagement (Bauman et al., 2012). In Canada, for example, it was found that 27% of individuals who engaged in leisure time sport in the previous 12 months reported running, with running found as one of the most popular sports across diverse cultures and regions (Hulteen et al., 2017). Promoting recreational running may help individuals obtain the health benefits of an widely accessible physical activity.

Despite initiating change in physical activity, individuals often do not maintain this behaviour in the longer term. Obtaining the health benefits of physical activity requires sustained performance of the behaviour, but discontinuation is common including for recreational runners (Bertelsen et al., 2017). In a systematic review of 56 studies examining correlates of recreational running, the reasons for discontinuation were varied, with injury and lack of motivation suggested as the main factors (Bertelsen et al., 2017; Fokkema et al., 2019; Menheere et al., 2020), whereas self-esteem and intrinsic motives were associated with sustaining running behaviour (Pereira et al., 2021). Effective strategies that support individuals in initiating and maintaining recreational running behaviour are required.

Habits have been theorised as a key process for behaviour change maintenance (Kwasnicka et al., 2016) and might be relevant in explaining physical activity behaviour change (Feil et al., 2021). One key element of habit formation is repetition, alongside stable contexts providing cues for the behaviour and reward schedules (Wood and Rünger, 2016). One study found repetition to be important especially during the earlier phases of habit formation and estimated a median of 66 days to form a habit, ranging from 18 to 254 days (Lally et al., 2010). Repetition may be accomplished by performing behaviour frequently, such as daily. A systematic review of goal-setting interventions for physical activity found daily goals to be associated with more effective interventions compared to weekly ones (McEwan et al., 2016). Strategies that support the performance of daily physical activity behaviours over a prolonged period such as approximately 2 months might thus support physical activity maintenance.

Behavioural ‘streaking’ is the engagement in a pre-specified behaviour consecutively in regular intervals such as daily without taking a break. Streaking is a frequently used strategy in recreational running (Barraclough, 2023). Run streaking is typically defined as running on consecutive days, for a minimum period or distance, typically at least one mile (1.61 km), any pace, any place. A run streak may be set for a period (e.g. specific number of days, weeks or months), or without an explicit end-goal in mind.

Streaking as a behaviour change technique has been suggested in lay audience-targeted self-help books (Clear, 2018; Guise, 2019). Lists and taxonomies that outline change techniques and strategies (Knittle et al., 2020; Kok et al., 2016; Michie et al., 2011) have not, to date, explicitly featured streaking as an option. Empirical evidence on streaking as a behaviour change technique has not been systematically captured in the health psychology literature. A better understanding of streaking might be beneficial to support habit formation in individuals considering long-term behaviour change.

The overall aim of this study is to examine streaking as a behaviour change technique in the context of recreational running behaviour. Specific objectives were to examine reflections on (i) the process of initiating run streaking, (ii) experiences of benefits and drawbacks of run streaking, (iii) the potential mechanisms through which run streaking might affect behaviour change including habit formation and (iv) features of run streaking that might transfer to supporting change in behaviours other than running.

## Methods

### Design

This study was designed using a pragmatic paradigm, intending to explore and generate hypotheses, and rejects a positivist onto epistemology and the quest to test hypotheses. The study used a qualitative approach using semi-structured interviews with recreational streak runners. Interviews were conducted online by an undergraduate student (MC) supervised by a health psychologist (SD). Interview length ranged from 18 to 56 min (*M* = 34 minutes). All interviews were recorded and subsequently transcribed and anonymised.

### Inclusion and exclusion criteria

Individuals were eligible for participation if they (i) were over 18 years of age, (ii) had been run streaking for a minimum of 66 days prior to interview (a length of time indicative of habit formation [Lally et al., 2010]), defined as running a minimum of 1 mile (1.61 km) every day and (iii) were recreational runners defined as athletes who receive no payment for their running activities. The study was conducted in English, with no restriction of geographical location.

### Recruitment and procedures

Recruitment was conducted via social media advertisement in two streak runners’ Facebook groups. Participants were purposively selected to include a range of ages, genders and streak lengths and were emailed an electronic document detailing the study and its methods. Verbal consent was received and recorded during the interview, which followed a pre-specified semi-structured topic guide assessing perceptions on running and run streaking. Given the relative novelty of the subject area, the topic guide was developed to capture general perceptions on running as well as questions about run streaking specifically focusing on initiating and maintaining run streaking, and elaborating on perceived consequences. Due to the study interest in habit formation, several questions specifically focused on habits, for example asking about running automaticity, routine and context (see Appendix A). This study received approval from the University of New Brunswick Research Ethics Board (approval code: REB 2020-169).

### Analysis

Interview transcriptions underwent abductive thematic analysis (Clarke & Braun, 2017) moving between inductive and deductive coding (Fereday & Muir-Cochrane, 2006); which allowed for the construction of unanticipated themes and knowledges as well as exploration through established psychological theory and concepts. Transcripts were first coded by one author using a deductive approach in line with the pre-specified research objectives based on broad general behavioural processes in line with psychological theory. Theory-based processes included the following:

Research objective 1 (initiating run streaking) was examined in line with social cognition models highlighting the intricate tasks required of translating motivation into behaviour.

Research objective 2 (benefits and drawbacks of run streaking) was examined in line with expectancy-value models (Wigfield et al., 2009) to provide a broad and general perspective on human behaviour resulting from expectant outcomes and the value placed on these outcomes. The particular emphasis in this study was on the subjective value individuals place on the outcomes of engagement in run streaking as a motivator for continued performance (Sheeran & Webb, 2016).

Research objective 3 (mechanisms of run streaking affecting behaviour change) was examined in line with intervention development frameworks (O’Cathain et al., 2019), which posit that theoretical variables are the mechanisms through which behaviour change techniques affect the performance of behaviour. The focus on mechanism paid particular attention to the concept of ‘habits’ given the alignment of streaking as a behaviour change technique with theorised conditions for habit formation such as repetition (Wood and Rünger, 2016).

Research objective 4 (transfer to other behaviours) was examined in line with multiple behaviour change approaches, which posit that performance of a behaviour may transfer to the performance of other behaviours (Fleig et al., 2015).

Within the broad inductive theory-based themes of initiation, experience, mechanisms and transfer, codes were generated inductively to identify additional themes. Coding included identification of additional themes, which were discussed by the research team, defined and refined. All codes were checked by a health psychologist (SD) to ensure that the transcriptions were consistently coded, as part of regular meetings during the analysis phase and discussed among the wider research team.

Interviews were conducted by a female undergraduate student (MC) who did not engage in run streaking at the time of interviews. This allowed participants to elaborate on their run streaking views and experiences in detail without assuming prior knowledge of the interviewer. The research team consisted of individuals who, at the time of the conduct and write-up of the study, identified as runners without engaging in run streaking (GO, GTG) and run streakers (NL, SD).

## Results

### Participants

**Figure 1.**
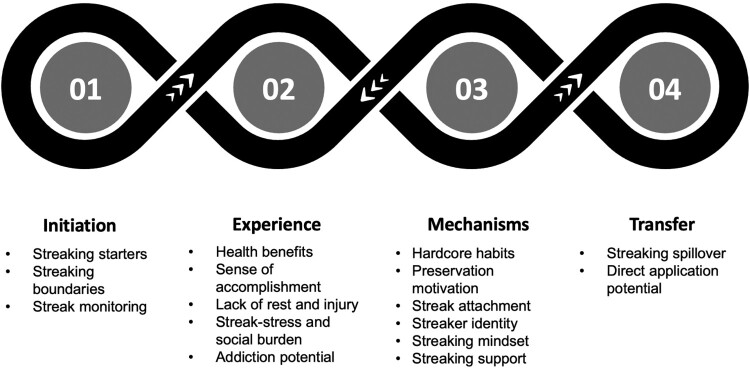
Themes identified in relation to the research questions.

Twenty-one individuals (11 females, 10 males) participated in this study. Participants reported streak lengths of 100–365 days (*n* = 3; all female), 365–500 days (*n* = 2; all female), 500–1000 days (*n* = 6; 4 female, 2 male), 1000–2000 days (*n* = 2; all male), 2000–3000 days (*n* = 5; 1 female, 4 males) and 3000–5500 days (*n* = 3; 1 female, 2 males). Two participants had previous streaks, which ended prior to their current one. Participants were aged 30–39 (*n* = 5), 40–49 (*n* = 11), 50–59 (*n* = 4) and 60–69 (*n* = 1). Based on self-reported weight and height, participants were classified as normal weight (*n* = 12), having obesity (*n* = 4), having overweight (*n* = 3) and not disclosed (*n* = 1). Participants reported their marital status as married (*n* = 11), single (*n* = 5), in a relationship/partnership (*n* = 3) or divorced (*n* = 2). This was an international study with participants from USA (*n* = 14), Canada (*n* = 2), Australia (*n* = 1), Italy (*n* = 1), Norway (*n* = 1), England (*n* = 1) and South Africa (*n* = 1). Ethnicities were reported as Caucasian (*n* = 13), Hispanic (*n* = 3), African American (*n* = 2), Caucasian and Hispanic (*n* = 1), Caucasian and Asian (*n *= 1) and African American & Latino (*n* = 1). Participants were either employed (*n* = 18), retired (*n* = 2) or unemployed (*n* = 1) (Table 1 and Figure 1).

**Table 1.** Participant demographics.

***ID***	Streak range (days)	Age range	Gender	BMI range	Marital status	Country	Ethnicity	Employment status
*1*	2000–3000	30–39	M	20–24.9	Single	USA	African American/ Latino	Employed
*2*	3000–5500	40–49	M	20–24.9	Married	Italy	Caucasian (Mediterranean)	Employed
*3*	2000–3000	40–49	M	20–24.9	Separated/Relationship	Australia	Caucasian (Australian)	Employed
*4*	100–365	40–49	F	30–34.9	Relationship	USA	Hispanic	Employed
*5*	3000–5500	40–49	M	20–24.9	Partnership	USA	Caucasian	Employed
*6*	500–1000	40–49	M	30–34.9	Single	USA	Hispanic	Employed
*7*	1000–2000	40–49	M	N/A	Married	Norway	Caucasian (European)	Employed
*8*	1000–2000	50–59	M	25–29.9	Married	USA	Hispanic	Unemployed
*9*	365–500	40–49	F	30–34.9	Divorced	USA	Caucasian/ Hispanic	Employed
*10*	365–500	30–39	F	20–24.9	Married	USA	Caucasian	Employed
*11*	500–1000	50–59	F	20–24.9	Single	Canada	Caucasian	Retired
*12*	3000–5500	40–49	F	30–34.9	Single	USA	African American	Employed
*13*	2000–3000	60–69	M	25–29.9	Divorced	Canada	Caucasian	Employed
*14*	2000–3000	40–49	M	20–24.9	Married	England	Caucasian (British)	Employed
*15*	500–1000	50–59	M	20–24.9	Single	USA	African American	Employed
*16*	2000–3000	40–49	F	25–29.9	Married	USA	Caucasian (Italian)	Retired
*17*	500–1000	30–39	F	20–24.9	Married	USA	Caucasian	Employed
*18*	100–365	30–39	F	20–24.9	Married	USA	Caucasian	Employed
*19*	500–1000	40–49	F	20–24.9	Married	USA	Caucasian	Employed
*20*	100–365	50–59	F	30–34.9	Married	South Africa	Caucasian	Employed
*21*	500–1000	30–39	F	20–24.9	Married	USA	Caucasian/ Asian	Employed

Note. BMI = body mass index (kg/m^2^), F = female gender, M = male gender.

### i) Process of initiating run streaking

There was diversity in past running experience; some participants had been running ‘*their whole lives’* prior to starting a streak, some had begun running with the start of their streak (‘*I haven't been running much more than my actual streak’*), with most falling in between these two opposites. Identified themes were ‘streaking starters,’ ‘streaking boundaries’ and ‘streak monitoring.’

#### Streaking starters

The reasons for starting run streaking varied and included ‪accidental beginnings, starting a personal challenge, a suggestion by someone or observing someone engage in run streaking, encountering streak running in a magazine or on social media, experiencing a life event or a combination of these factors.

For some participants, their run streak started organically without the goal of running every day to develop a streak. Those participants recounted retrospectively finding out that they had been doing ‘*a thing’* that others also practiced.

‪I remember the conversation I had with the guy […] I said: ‘Yeah, I do [run] every day’, and he goes: ‘Oh you’re on a streak?’ And I went: ‘What?’, […] ‘I don't even know what you're talking about.’ So I went back and looked it up and like oh, right. Yeah, that's me. Participant 3, male

Several participants reported a combination of factors coming together at a point in time which led to them starting their run streak.

‪It was January 1st [YEAR] and I think it was just a combination of things. I was just fed up with my sedentary lifestyle. At that time I was going through divorce. Needed something different. I needed something challenging and […] I just stumbled across this streaking thing, and I thought […] ‘Well, I can do that’. Participant 4, female

For some participants, the decision to start run streaking was intentional and they could recall the date when they started their run streak.

It's life changing in a weird way, it's like a life changing kind of experience. I can still remember that day going out, [date] and running and being like, well, this is going to be interesting and then the fact that I got through a whole year and now it's 12 and a half years later and it's still going. Participant 5, male

#### Streaking boundaries

Participants reflected on several topics in relation to performance boundaries set around their run streak in relation to number of days.

In terms of the number of consecutive days, participants often reported dissolving or shifting boundaries, where they started streak running for a period and then ‘*just kept going*’ once the initial goal had been reached.

Once I got to 100 [days of running consecutively I] just kept going and just wanted to keep going wanted to keep working on my health, wasn't happy where I was health wise. And so I just wanted to keep going with it. Participant 6, male

Several participants noted that they set new goals as their streak evolved, looking for new milestones.

I was like okay well if 30 days is awesome. Let me see if I could do 100 days, and then saw that like that became the new goal. And then once I got to the 100 days I was like, alright, I'm gonna do the whole year because that just seems crazy. Participant 18, female

Several participants aimed for streaking milestones which included 30, 100, 365 days (and subsequent yearly anniversaries), 1000 days (also known as ‘Comma Day’ as the quadruple number is often written with a comma) or around 1170 days (also known as ‘Forrest Gump Day’ after the number of days Forrest Gump runs consecutively in the movie with the same title), and many further milestones to be reached throughout.

I had completed a year, so I thought, well, let's see how far I can get. And another year past. And then I was like, OK, well I'm going to try and hit the Forrest Gump, and so I hit that. Participant 16, female

Some participants noted that they deliberately do not set ambitious goals and observe the incremental progression of their run streak.

I did this seven days and then after that I'm like well might as well go for week number two. I never set a goal like I'm going to do it for one year. It was always like almost like increments. Participant 11, female

In terms of daily distance, streaking is typically defined as running a minimum of one mile within a day, to which all participants had subscribed as the minimum criterion for keeping the streak going. There was variation in terms of the additional goals set. Some participants kept the one mile goal as their minimum target. Others set additional or higher goals, typically expressed as distance run, or in some cases time per day ran.

I do like to try and make sure that bare minimum I’ve done a 5K at some point during the day and I think that works out quite well […] As long as I can find 25 minutes is all that's needed to make sure I've done the 5K. Participant 14, male

‪Several participants reported having done or doing a ‘streak within a streak’, whereby they set a higher minimum distance or time and additionally monitored the number of consecutive days of achieving this distance.

I’ve been running, trying to run, 10, or more miles a day. So, I’ve been doing that since [date], I don't know when I'm going to stop but that's my streak within a streak. Participant 5, male

#### Streak monitoring

Given the ongoing goal to ‘*keep the streak alive*’, all participants reported engaging in some form of self-monitoring. There were five methods that were specifically described in interviews. Streak runners used one or more of these methods to help track their streak: fitness watches, journal/calendar, social media, spreadsheets and smartphone apps.

I have a spreadsheet which is so ridiculous […] so usually once a year or couple times a year I'll just like check the mileage and put it in the spreadsheet. Participant 12, female

Some apps mentioned were Strava, MapMyRun and Run Keeper. Facebook groups and social media services like Strava were seen to produce the social support alongside public monitoring.

### ii) Benefits and drawbacks of run streaking

Participants noted several benefits of streak running which were categorised *as ‘health benefits’* and ‘*sense of accomplishment’*.

#### Health benefits

All participants noted health benefits gained from run streaking which covered several components of health, including psychological, physical and social aspects. Multiple health components were mentioned in participant statements, demonstrating their interconnectedness.

It [streaking] just made me feel stronger physically. My body has changed and altered. So I feel better that way. And I'm able to keep the weight off so I see physical changes as well as feeling emotional changes and mental changes. Participant 16, female

The most frequent health benefits mentioned were psychological health improvements, with streak running described as a ‘*stress relief*’, ‘*self-care*’ and a chance to ‘*decompress*’. Participants noted how the daily run was an opportunity to disengage from other tasks and ongoings, to engage in reflection, or step away to disengage and switch off. A few participants described not being fully aware of the psychological benefits whilst engaging in streak running but noticed these when their streak ended.

I've realized that it [streaking] has a benefit for my mind that I wasn't aware of. And I would just find myself getting so uptight. And I think: ‘Well, when can I think about this or when can I deal with this, I don't have time’. And then I realized that a lot of that stuff I did while I was running. Participant 20, female

Other participants speculated that the streak running might have had a preventative impact on their psychological health and wellbeing.

I’ve never turned to running to solve mental health problems, but I can't help feeling it’s had a preventive impact. Participant 2, male

In addition to psychological benefits as outcomes, streak running was frequently described as pleasant in itself, with participants stating that they ‘*love to run’*, and that running is something that induces joyful anticipation.

[streak running is] something I look forward to every day, whether it's 100 degrees and 100% humidity outside. Or minus three or four and cold as heck and snowing. We [streak runners] like to get out and run. It’s just fun. Participant 8, male

In terms of physical health benefits of streak running, participants noted feeling ‘*active*’, ‘*healthy*’ and ‘*fit*’. Several participants reported weight loss and weight (loss) maintenance as a result of having started streak running.

When I started [streak] running I weighed almost 200 pounds. I'm not far from that. Still I'm at 160, but I've managed to keep my weight down. Participant 16, female

However, weight change was not universally experienced by everyone, with one participant referencing compensatory eating behaviour as an explanation.

It doesn't make you lose weight. I could tell you that much, ‘cause then I go eat a burrito after. Participant 12, female

In terms of social health benefits, participants noted two ways in which streak running allowed them to connect socially in positive manner. The first way was connecting to other runners, especially streak runners. Participants reported perceiving a sense of community by having the option of connecting with other streak runners, often through social media groups which provide a forum for social exchange and support.

It [streaking] is one of those things where you can be part of a very large community and a big group, and at the same time still have your solitude. It's an individual sport that is also part of a very large community and the amount of support you get is really huge. Participant 4, female

The second way was through social feedback from others about streak running, which often included positive comments, admiration, and other statements of appreciation.

I've had a lot of people say, how they like look up to me, or that they find it inspiring that like, you know, no matter what, you can continue that. So I mean, I find it special. Participant 9, female

#### Sense of accomplishment

Participants frequently noted that run streaking gave them a feeling of achievement, noting that their run streaking made them feel ‘*proud*’, ‘*kind of superhuman*’ or provided a source of ‘*satisfaction*’.

I’m quite proud of myself, it gives you a sense of accomplishment. It's just you that did it. Participant 20, female

Participants reported two levels of accomplishment. The first level of accomplishment was based on daily goal completion, with participants noting the recurrent feeling of achievement by accomplishing the daily run.

And then, it also gives me that satisfaction after it's done for that day. It's that great satisfaction, like OK, another day checked off. Participant 15, male

The second level of accomplishment was more reflective when participants noted the additive nature of their accumulating streak days, which became a point of pride with increasing streak length.

You know, that kind of sense of accomplishment that you get, which is huge. Wow, I did this really, you've run every day for so long. Participant 20, female

Some participant accounts were indicative of the sense of accomplishment led to a positive affect towards the streak.

So it's uh to be this far in now it's almost a point of pride that I’m not going to stop. Participant 17, female

The reflective sense of achievement could be accompanied by strong emotive states which often clustered around accomplishing streaking milestones, such as a round streak day number or at the end of a calendar year, or a personal birthday.

I've cried after I finished some of my streaking days where I've accomplished like my 2000th run streak day [...] I'm like there you go 2020 [the year the COVID-19 pandemic broke out] I just beat you. So yeah, so like it just gives me a sense of accomplishment. Spiritually, mentally, emotionally. Participant 16, female

Other reflective senses of accomplishment were in the context of social commentary, where non streak runners remarked on the streaking accomplishments.

And like when I tell people who don't run about you know that I run every single day like they like I don't think they even get it at first. And it just makes me feel kind of superhuman which is really cool, I like that. Participant 18, female

Some reflective accounts of accomplishment were situated within the context of a person’s life history. Narrative accounts placed reflections of run streaking alongside the unfolding of life events, with ‘the streak’ almost akin to a friend or partner who was present through time.

I’ve moved, I’ve lost parents, lost cats. I've moved again, gone through a few jobs. The consistency has been, I've been running, it's amazing, like I turn 50 in a couple days and I'm like, God, I've run every single day of my 40s, every single day, it's amazing you know, it's fascinating to me, it's a great experience, something I’ll always cherish. Participant 5, male

Participants also reflected on negative consequences experienced throughout run streaking. Common drawbacks identified included ‘*lack of rest and injury’*, ‘*streak-stress and social burden’* and ‘*addiction potential’*.

#### Lack of rest and injury

Participants frequently reported experiencing injuries, which were extended or aggravated due to continuing streak running. Injuries were seen as induced by streak running for some participants, particularly due to a lack of rest and recovery.

I have a hip issue that I've been having for a few years and not giving my body the rest that it needs, probably long-term effects, I may end up needing a hip replacement. Participant 16, female

To preserve their run streak, many streakers reported enduring pain and some reported furthering their injuries.

… what should have been, I think a 2 1/2-week recovery ended up being 110 days recovery from this operation and I continued to run throughout that. Participant 14, male

Injury management strategies included persisting and enduring the discomfort and pain (‘*ran with a broken toe*’), slowing down the running pace and reduce milage to the minimum requirement of one mile to ‘*keep the streak alive*’.

This summer for about three, four months my achilles was just really grumpy. And I know from years of running that the best remedy was just to stop running. But being a streaker, […] I'm gonna keep streaking, I'm just gonna cut my mileage, just run a mile, just to get my streak and just keep the streak going and get the minimum that you need. Participant 6, male

Some participants who got health professional advice reported ignoring advice that was not in line with their ability to continue run streaking, or seeking different health professional advice which was in line with their goal to keep streaking.

I saw a physio and they said: ‘you got to stop running. You need rest’. And I'm like: ‘I can't do it’ […] So I went and I looked up this sports physiologist. I went [and] I said ‘I'm a streak runner’ and he goes: ‘I get that’, he said: ‘If I was to tell you to stop running to fix your hamstring you're then going to have to go somewhere else to fix your head. So let's come up with something that works. Keep your streak running’. Participant 3, male

However, not every streak runner reported injuries.

I’ve been doing it for more than eight years and never got injured. It's the body's learned to cope with me. Participant 2, male

#### Streak–stress and social burden

There were many instances of participants explaining the stress that accompanies maintaining a run streak. Often the streak–stress was felt when usual routines and running times were disrupted, either due to unforeseen changes in the schedule or due to special events such as holidays and vacations.

I would say sometimes I get a little anxious if like, I haven't ran, like, vacation. Participant 9, female

Participants reported that streak-stress increased over the course of a day when the goal of the daily run had not been met, and started to occupy their thoughts, with participants wanting to get the streak run completed so that they would not have to think about it.

If I'm driving to work and I've not gone out in the morning, I do get bit […] clockwatching. Participant 14, male

Pre-occupation and prioritisation of run streaking was reported to have social implications for some participants, for example preventing spontaneous social gatherings and other social events.

Especially like Christmas or New Year's where typically people stay up late […] I'm thinking: ‘No, I can't do that. I gotta get up early tomorrow for my streak’. […]’ So I do feel that it has impacted a lot of things of my life. Participant 6, male

Participants often reported that individuals who were not (streak) runners would question the rationale for streak running and ‘*don't get it*’. Some participants reported that the streak was seen as a burden by spouses, family and friends, and could be perceived as a self-indulgent activity.

It can be seen as a selfish act by the other person’s significant other. I have known people whose spouse or partner has been resentful of their streaking. I knew a friend of mine, his wife would even kind of sabotage his streaking. Participant 4, female

#### Addiction potential

Streaking was perceived to have addictive properties by some participants. Some mentioned substituting health-impairing addictions, such as alcohol and drugs, for streak running.

So, I think what I've done is somehow managed to swap a bad lot of addiction to a less bad addiction. Participant 3, male

Streak running was described by some as an obsession rather than an addiction, and they noted that it was a more health-promoting behaviour, compared to other possibilities.

I think I've seen some people get obsessed by it. But I would suggest that maybe those people would be obsessed by something. And so maybe streak running is a healthier obsession than many other choices, and so if it fulfills their need to be obsessed, that's also great. Participant 7, male

There were generally two types of streakers: the determined streaker where the streak is viewed as something they must do with absolutely no exception, and the flexible streaker, who reported to be able to end the streak when an injury or another barrier prevented them from running.

It’s a commitment, it's not an obsession. It's not an addiction. It's, those terms have negative connotations to them. If I wanted to stop, I could. I don't have any issues with that, I just really enjoy getting out and going [running]. Participant 8, male

### iii) Mechanisms of run streaking

#### Hardcore habits

Most participants reported high levels of automaticity of their running behaviour indicative of the presence of deeply engrained running habits.

I can't remember the last time I run wasn't first thing when I got up, because that's just, it's so automatic. Participant 3, male

Several participants reflected on the low cognitive effort they needed around their run streaks.

Whether it's maybe right after work at 5:00PM or at 11:45PM, it's just it's second nature. I don't even think I just start subconsciously getting my running clothes on, getting out the door again a mile in just coming right back. Participant 1, male

There were a variety of contextual cues mentioned which prompted the initiation of running behaviour such as waking up in the morning, children going to sleep or to school, or getting home from work. In many cases, the routine of streak running had been built into the patterns of daily living.

On days that [wife] works during the week, during the day, we both get up in the morning, so we run at 4:00 o'clock [in the morning] and one goes. The other comes back, the other goes and we try to get our runs in before the baby wakes up. Participant 8, male

Many participants also described ‘*openings*’ or ‘*windows*’ during their days which were seen as opportunities to get out for the daily run. Identification of these opportunities was seen as effortless.

Whether I run in the morning, in the evening in the afternoon. It's just part of my day. I don't really have to think much about it. Participant 6, male

During times of disruptions in their daily routines and context participants reported being less able to rely on their running automaticity. These disruptions could come from vacation, changing work schedules, or pandemic-related restrictions. Many streakers were flexible and able to cope with these.

Most of it is automatic. Planning is more so like if I'm traveling or if I know like I have a really busy schedule. You know, like if I have a day that I have a lot of meetings or you know a lot of stuff like that. I have to be very like intentional. Participant 12, female

These automaticity disruptors reported by participants suggest that other mechanisms are present in addition to habits and automaticity, to maintain the run streak. Additional mechanisms identified were ‘*preservation motivation’*, ‘*streak attachment’*, ‘*streaker identity*’, ‘*streaking mindset*’ and ‘*streaking support’*.

#### Preservation motivation

Most participants reported a strong motivation to ‘*keep your streak alive’* as a motive for their running behaviour. Their streak related motivation ensured performance of running behaviour including ‘*days where you don't feel like running*’ and motivation to run was low. The motivation to run was outsourced away from the behaviour itself towards streak maintenance.

You know, some days I physically do not want to run. I just want to lay on the couch and just do nothing, but because I've gotten so far, I feel like I have to keep going, so maybe that's a part of it. Participant 12, female

Often, the motivation to maintain the streak was fueled by anticipated regrets of how one would feel if the streak broke, and a fear that a broken streak would lead to a decline in running behaviour.

I think I'm so happy with the effects of running that I don't ever want to break that streak ‘cause I fear if I broke the streak, that might be the start of the beginning of the end or falling apart. Participant 3, male

Many participants recalled the ‘*tough days*’ and ‘*days you don't want to*’ and ‘*just don’t feel like going out to run*’. Momentary motivational lows were overcome through streaking, and refraining from going for a run on these days was presented as not being an option, because ‘*you have to do it*’. Participants often referred the ongoing streak holding them accountable and committed to persist, providing a reason for consistency.

‪The consistency is what helps me stick with it. I know that if I didn't run tomorrow then I probably wouldn’t run another day in a couple days. And then it's just kind of a cycle. So the streak I think the consistency of it keeps me on track and dedicated to being active. Participant 21, female

#### Streak attachment

Most participants displayed a relationship to their streak akin to having an attachment with a person, pet, or a desired object. The common phrase ‘*keeping the streak alive*’ indicates viewing the streak as more than a behaviour change technique. This personification or objectification was often illustrated in participants recalling stories and experiences made together with their run streak. These included accounts of having ‘*done a few crazy things to keep it going*’ stories, such as running in car parks, airports or hotel rooms.

I’ve done weird runs around like my living room couch, […] I wasn't able to access a treadmill and it was a blizzard. I’ve run around couches. I’ve run in my backyard like just laps when I couldn't leave my kids home alone. The weird things […]. Participant 17, female

Several participants recounted seeing ‘*beautiful places*’ which they would not have encountered without streak running.

I was running in Amsterdam early in the morning and you get to see a side of the city that most tourists don’t see […] that's been a big thing for me is this really interesting places that I've seen from a completely different angle. Participant 20, female

These unique experiences whilst streak running often seemed to deepen the attachment to the streak by creating positive associations and conversational content for social contexts.

I’ve done a few crazy things to keep it going. I think most streakers will tell you they've done a few different things […], but it feels like fun as well. It feels a bit special, it's the way to be crazy without being crazy, but I think all of us have a little bit of a need for. Participant 7, male

#### Streaker identity

All participants reported identifying as a streaker indicative of identity driving engagement in streaking as a behaviour change technique. Being a streaker meant that running every day was a duty, with strong rejections of the possibility of not engaging in a daily run.

‪I consider myself a streaker. […] But if I literally cannot run, that’s what it would take. If I just for whatever reason, could not bring my legs to move faster than you know my walking, actually be considered running, that's what it would take [to stop running]. Participant 16, female

#### Streaking mindset

Streak runners often reported a switch in their outlook on the daily streaking task. One participant remarked on a change in mindset when the question changed from a motivation focus of “will I run today?” to an action focus as to “when will I run?”. This shift in mindset was commonly reported suggesting that implementation of the goal to run every day is the primary cognitive occupation, rather than deliberation as to whether to run in the first place.

And I found that the [marathon] training was […] hard. To say I'm going to do it four times a week for me became very challenging because there was always tomorrow. There was always other priorities […]. And the streak means that there's no longer a question, or the question is no longer if I will run, its when I will run. Participant 7, male

The daily nature of streaking seemed to prevent participants from having to decide whether to run on a particular day, as the decision had already been made by committing to run every day. Some runners saw streak running as taking less effort than intermittent running, suggesting that the cognitive effort to decide whether to run on a particular day might outweigh the physical effort to run daily.

I don't have the discipline to do three four times a week, it's far easier for me to go every day. Participant 2, male

Some participants remarked that the daily nature decreased internal self-talk which can result in non-performance, as one is committed to the behaviour regardless of transient adverse contextual factors, such as unpleasant weather conditions.

I'll always find an excuse to not do something. Oh I can do it tomorrow and you see if you're streaking there is no tomorrow You have to do it so if it's snowing or raining. You have to just run. Participant 20, female

#### Streaking support

Support from family, peers, and other streak runners contributed to the overall motivation to continue the behaviour. In addition to exercise specific social media platforms such as Strava, many streakers were active on general social media channels with interest groups specific to streak running, sharing their streaking progress, and following the journeys of others. Moreover, the social nature of sharing accomplishments was often reported to function as a source of motivation for one’s own ‘*streak journey’*.

… and then seeing these people like I just finished 40 years you're like, Okay, something maybe to aspire to. Participant 5, male

### iv) Streak transferability features

Participants reflected on two ways as to how run streaking might transfer to other areas of life which were identified as ‘*streaking spillover’* and ‘*direct application potential’*.

#### Streaking spillover

The first was a general reflection on ‘*streaking spillover’* effects, including the lessons learnt and skills acquired through streak running and how these might apply to other areas of life. Spillover effects included general high level life lessons.

‪It's also something that matches my philosophy, if you like. My general motivation is that big things come one step at a time […]. It's about the streak. You know it's many days now, but it's just one day at a time. Participant 7, male

Other streaking spillover effects were cognitive transfer, whereby positive beliefs and emotions were applied to other areas of life.

I’ve gained confidence with just feeling like physically I could do about anything I set my mind to because streaking is not easy by any means. Participant 16, female

A further streaking spillover effect was behavioural transfer, whereby the goal of daily streaking was reported to impact on other behaviours which were seen as related to streak running, including sleeping, eating, and alcohol consumption.

[I am] more calm, maybe more conscious about what I eat, conscious about being hydrated. Knowing that if I want to have a good run the next day, I don’t want to feel ill or maybe upset stomach or whatever. Participant 6, male

#### Direct application potential

The second reflection on streaking was ‘*direct application potential’* of the strategy of streaking to other areas of life. Most participants noted that streaking as a technique has the potential to be used to change behaviours other than running.

Streak running helps you out. Teaches you structure and discipline to where you might want to take on another task. Oh maybe I can start being consistent on task ABC or Task XYZ ‘cause you have the ability to keep your running streak going. What else can I do consistently throughout my life? Participant 1, male

Although not every participant used behavioural streaking to change other behaviours, several participants mentioned attempting a streak including daily push-ups, journaling, meditating, walking 10,000 steps a day, warming up prior to exercise, completing work tasks, biking, and learning a new language.

I wanted to see if I can try to read […] a couple of pages like learn something new like a new aspect of the programming language that I know now, or learn a new language, not the whole language in a day, but like you know, just trying to continuously learn like streak learning. Participant 1, male

One participant reported being engaged in a push-up streak (on day 250 at the time of interview), but overall there were limited instances of participants reporting successful use of streaking for other behaviours similar to the streak they developed for running.

I've done like writing in my journal as a streak I guess like trying to do that every day. That wasn’t so successful. Participant 5, male

Some participants speculated as to why the direct application potential of streaking remained unfulfilled, including a lack of momentum and lack of social support and community.

I did keep track of them, but I just didn't feel like the sense of pride that I think ‘cause there's not exactly a community or Facebook groups based around on, you know, streaking for meditation or streaking for reading a book. Participant 4, female

## Discussion

### Principal findings

Streak runners reported a variety of reasons leading to the starting of a run streak, including unintentionally starting, engaging in challenges or life events such as divorce or changes in health status. Boundaries around the run streak varied in terms of daily distance with all participants adhering to the daily goal of one mile as the minimum requirement to ‘keep the streak alive’. All participants monitored their streak length in some way.

Streak runners reported a variety of physical, mental and social health benefits of streaking. Streak running provided an ongoing and growing sense of accomplishment as daily goals are met and the streak length increases. Streak runners showed an emotional attachment to their streak as an inherent part of their daily life. Drawbacks of run streaking included injuries and a lack of rest and recovery with many streak runners reporting running with injuries or aggravating these. Streaking could lead to periods of stress and burden particularly when daily routines were disrupted. Some noted that streak running had addictive properties for them, but others did not perceive this.

Reflections on streaking mechanisms were in line with accounts of automaticity indicative of habitual behaviour. Other potential mechanisms identified as leading to maintenance of running behaviour were motivation with streak runners reporting to be highly motivated to continue their streak, including days when they were not motivated to run. Streak runners reported a strong level of attachment towards their run streak and all held the identity of being a ‘streaker’, leading to persistence in daily running. Streak runners reported a mindset shift focusing on opportunities to run, rather than reflections on whether to run on any given day. Streaking as an activity was reported to receive social support from friends and family as well as other run streakers, often virtually through social media. However, social support was not universally reported.

The positive effects streak runners perceive often spilled over into other areas of life, including increases in self-esteem and improvements in other health behaviours. Streaking as a general behaviour change technique was seen as having potential to be applied to other behaviours, with participants reporting some applications of this technique to other areas of their life.

### Strengths and weaknesses of the study

This study examines streaking as a real-world behaviour change technique which has received relatively little research attention to date. The international nature and balance of genders serve as strength to the study. Overall, this study offers in-depth recounting of experiences which offer insight into the naturally occurring phenomenon of behavioural streaking in the context of recreational running. Limitations were experienced as there was an abundance of willing participants, but selection was narrowed due to the qualitative nature of the study. Furthermore, the study does not have representation of the younger demographic of run streakers, and participants came from high-income countries only.

### Relation to other studies

Behavioural streaking is currently not included as a behaviour change technique in existing taxonomies and lists (Knittle et al., 2020; Kok et al., 2016; Michie et al., 2011). A behaviour change technique has been defined as ‘an observable and replicable component designed to change behaviour. It is the smallest component compatible with retaining the postulated active ingredients and can be used alone or in combination with other BCTs’. p. 2 (Michie et al., 2015).

The current study suggests that behavioural streaking is an individual technique with two essential components. The first component is goal setting, specifying a minimum criterion for engagement in the target behaviour. In the context of run streaking this was running a minimum distance (typically one mile) during a 24-hour time window for consecutive days. Some streak runners pre-specify the number of days they intend to achieve, often in the context of challenges (e.g. 30-day challenge), but most follow open-ended daily goals. The second component is self-monitoring, by counting the number of consecutive units of engagement, typically days as in the context of the current study. Streaking as a behaviour change technique requires both components to function as a technique; the goal with a minimum criterion for accomplishment and the ongoing monitoring of consecutive units of engagement, e.g. daily. Behavioural streaking cannot be broken up into smaller constituent components without losing its ‘active ingredients’ and can thus be considered a behaviour change technique in itself.

Given that streaking is an organically occurring technique that individuals use, no mechanisms of action through which this technique operates have yet been hypothesised. The current study suggests that streaking might be a useful technique to develop behavioural automaticity indicative of habits (Gardner, 2015). Given the repetitive nature of performing the behaviour daily, habit theory would suggest the development of automaticity in response to stable or recurring contextual cues. Moreover, participant accounts of feeling a sense of daily accomplishments and a sense of pride when reflecting on the growing number of streak days reflect the reinforcing and rewarding nature of using this technique. Streaking thus fulfils essential components suggested by habit theory – repetition, contextual cues, feelings of reward – suggesting it may be a useful technique to support habit formation if used for a period of time, such as around 2 months (Lally et al., 2010).

In addition to automaticity, streak runner’s reflections on their streaking were indicative of other mechanisms through which streaking might lead to behavioural performance. These mechanisms include motivation and goal setting, identity, self-regulation including coping planning, social support, and self-esteem, and are in line with theoretical processes of behaviour change (Michie et al., 2005). Moreover, streak running seemed to lead to the frequent use of additional behaviour change techniques such as action planning and coping planning, in instances where run streakers required self-regulation to implement their goal of daily running and at times where emerging barriers interfered with their typical running routines.

Evidence suggests that self-esteem and intrinsic motives are associated with sustaining running behaviour (Pereira et al., 2021). The current study supports this finding, with streak runers reporting a high level of intrinsic motivation for running behaviour, and a high sense of accomplishment and a sense of self-esteem resulting from repeated goal achievement. The current study extends the motivational findings, suggesting that streaking as a technique may facilitate situations where in-the-moment behavioural motivation is low, by outsourcing motivation from the behaviour towards streak maintenance. This switch from intrinsic motivation to ‘preservation motivation’ helps the individual to overcome moments and periods where performance of the behaviour itself is undesirable in the service of achieving the goal of not breaking the streak. The conditions under which ‘preservation motivation’ might operate are with increased streak length and growing emotional attachment to the streak as something to retain and keep going.

### Implications and future research

Streaking may be a promising behaviour change technique to support habit formation in running behaviour, and potentially other behaviours. The current study examined experiential accounts of individuals who had used streaking for an extended period. A key question remains of whether the technique may be used to help individuals start and maintain a new behaviour. Moreover, the mechanisms through which behavioural streaking might operate, particularly motivation and goal setting require future attention.

One main drawback of run streaking is the potential to cause or aggravate injuries, which evidence suggests is one of the main reasons for novice runners to cease running (Bertelsen et al., 2017). This suggests that the use of streaking as a strategy needs to be prudent, and running distances will need to be built gradually, potentially with a pre-streak period. Further questions remain regarding who might benefit from streaking in terms of individual difference variables, and whether this technique might be more suitable for certain individuals and within certain living contexts. Moreover, given the strong sense of identity, emotional attachment to the streak it would be important to further understand the potential adverse consequences of a streak breaking.

Future research should also focus on the transferability of behavioural streaking to other behaviours which can be performed in regular intervals such as daily. Run streakers in the current study engaged in daily running for long periods of time, and it is important to establish whether the appeal of behavioural streaking for some is restricted to the unique nature of running behaviour, or if behavioural streaking can also apply to other behaviours. In general, streaking might be more suitable to certain types of behaviours than others. Lastly, given the importance of social support for engagement in this behaviour change technique, and some suggestions of social tensions withing the streaking accounts in this study, future research might focus on understanding the social factors associated with behavioural streaking.

## Conclusion

Streaking is a behaviour change technique with potential to support the formation of behavioural automaticity indicative of habits, as examined in the context of recreational running. Future research needs to study this promising, organically occurring technique to understand its potential to support long-term behaviour change.

## Supplementary Material

Look over there a streaker_Topic guide.docx

## Data Availability

The data that support the findings of this study are available from the corresponding author upon reasonable request.
