# Supplementary material for: Look, over there! A streaker! – Qualitative study examining streaking as a behaviour change technique for habit formation in recreational runners
Source: Health Psychol Behav Med. 2024 Oct 21;12(1):2416505. doi: 10.1080/21642850.2024.2416505 (PMC11494719; doi:10.1080/21642850.2024.2416505)
Supplement: Look over there a streaker_Topic guide.docx [file RHPB_A_2416505_SM4729.docx]

Appendix A

**Interview Guide – Streaking Study**

**Introduction:**

*Thank you for volunteering as a participant for this research study. This interview should take around 30-45 minutes, but might be a little bit longer depending on the length of your answers. I will now go over the information letter that was previously sent out via email.*

[go through information letter].

*Do you consent to participate in this research? Do you acknowledge understanding and agree to the information outlined in the participant’s information letter?*

**Questions:**

1. **For how long have you been running?**
2. **How much of that have you been streaking, and what’s your streak number?**
3. **Do you consider yourself to be a ‘streaker’?**
4. **Do you know many other people who streak?**
5. **How did you get into streaking?**
   1. What made you want to start a streak?
   2. Accidental or planned?
6. **How did you decide on the boundaries of your streak?**
   1. minimum distance?
   2. Minimum time?
   3. upper limits?
   4. Rules for breaking the streak?
7. **How do you monitor your streak?**
   1. App
   2. Journal
   3. Memory
8. **Have you ever done a streak within streak (running minimum of a mile, but also counting the days of running 7 miles consecutively)?**
9. How has the COVID-19 pandemic affected your streaking?
   1. Difficult or easy to keep streaking
10. **What does streaking give you?**
    1. What do you think is special about streaking?
    2. What do you get from streaking?
    3. What changes in your life have you seen since streaking?
    4. Are you involved with any social groups or organizations around streaking?
11. **Are there any negative consequences of your streaking?**
    1. injury
    2. missing out on social occasions,
    3. time and energy investment

*In the next section I would like to talk about habits and streaking as a strategy for changing behaviour.*

1. **Would you say that your streaking is something which you do automatically without thinking much, or do you still have to plan when to do it?**
2. **What is the context in which you streak?**
   1. Place?
   2. Time of day (Morning, noon, evening, night)?
   3. Time in the week (weekday vs weekend)
   4. Prior action?
   5. Triggers?
3. **How is your streak affected when there are disruptions in your daily routine?**
4. **What would it take to stop you from streaking?**
   1. Injury
   2. Surgery
   3. Life events (e.g. sick partner, kid or relative)
5. **How would you feel if you had to stop your streak?**
6. **If you ever had to stop, would start again, once you can?**
7. **Have you used streaking in other areas of your life?**
   1. eating
   2. alcohol
   3. tidying up
8. **What lessons could people who want to change their behaviour learn from streakers?**
   1. What did streaking teach you about changing your behaviour?
9. **Is there anything else about streaking that you want to tell us that we haven’t asked?**

*I would like to ask you a few demographic questions to finish off.*

1. What is your age?
2. What is your sex? (male, female, other, prefer not to answer)
3. What is your marital status?
4. What is your weight and height?
5. How do you describe your ethnicity?
6. What is your employment status?
7. Do you want us to send you a summary of the findings of this study?

*Thank you for participating in this study.*
